# The Effect of an Optimized Wet Milling Technology on the Crystallinity, Morphology and Dissolution Properties of Micro- and Nanonized Meloxicam

**DOI:** 10.3390/molecules21040507

**Published:** 2016-04-21

**Authors:** Csilla Bartos, Piroska Szabó-Révész, Csaba Bartos, Gábor Katona, Orsolya Jójárt-Laczkovich, Rita Ambrus

**Affiliations:** 1 Department of Pharmaceutical Technology, University of Szeged, Eötvös u. 6, H-6720 Szeged, Hungary; bartoscsilla@pharm.u-szeged.hu (C.B.); revesz@pharm.u-szeged.hu (P.S.-R.); bartoscsaba@pharm.u-szeged.hu (C.B.); katona@pharm.u-szeged.hu (G.K.); laczkovo@pharm.u-szeged.hu (O.J.-L.); 2 Gedeon Richter Plc., Gyömrői út 19-21, H-1103 Budapest, Hungary

**Keywords:** wet milling, micronization, nanonization, physical structure, crystallinity, *in vitro* dissolution

## Abstract

This article reports on the effects of a new combined wet milling technique on the physicochemical properties of meloxicam (MEL). The influence of milling time on the particle size, the crystallinity, the morphology and the dissolution rate of MEL has been studied in the presence and absence of polyvinyl alcohol (PVA) as a stabilizer agent. Micronized MEL particles were produced in aqueous medium which did not contain additive after milling for 10 min. For nanonization an additive and longer milling time were required. After particle size determination the structural and morphological characterization of the wet milled, dried products containing MEL were studied. X-ray powder diffractometry (XRPD) and differential scanning calorimetry (DSC) examinations revealed the change in the crystallinity of MEL. Scanning electron microscopy (SEM) images showed that aggregates of nanosized MEL particles were formed, regardless of the presence of PVA. The nanonized MEL crystals (D_50_ = 126 nm) exhibited a regular shape and a smooth surface. The increased specific surface area resulted in a high dissolution rate and concentration of free MEL. According to the results, the produced samples could be applied as a basic material (micronized MEL) and intermediate product (micronized and nanonized MEL with PVA) for the design of dosage forms.

## 1. Introduction

One of the major current challenges of the pharmaceutical industry is related to strategies that improve the water solubility of drugs because over 40% of new drug candidates are water-insoluble compounds [1]. Poorly water-soluble drug properties can impede the effective delivery of these drugs into humans, and affect their dissolution rate and subsequent absorption at the site of activity [2].

Poorly soluble molecules have been successfully formulated by employing a variety of techniques to modify the physico-chemical and biopharmaceutical properties of drugs [3,4] such as: (i) solubilization in surfactant solutions; (ii) use of co-solvents [5], salt formation [6], complexation with cyclodextrins [7], crystallization [8], amorphization [9], milling [10], *etc.*

Milling is a technique commonly applied to produce micro- or nanosized drug crystals in order to increase the dissolution rate and absorption, and hence the bioavailability of poorly-soluble materials. According to the Noyes-Whitney equation, the reduction of the particle sizes of drug crystals increases the specific surface area, which can improve the rate of dissolution of the drug [11]. There are many different well-known types of milling techniques with both advantages and disadvantages; dry and wet milling can be distinguished [12,13]. Wet milling requires less energy and time than dry milling. Thanks to the environmentally isolated system, it is a dust free process and the material is less heated up [14]. However, wet milling has some disadvantages as well, e.g., increased wear of the grinding medium, corrosion hazards, *etc.* To overcome the limitations of the conventional particle size reduction technologies for poorly-soluble drugs, new combinational methods have been developed for the production of ultra-fine suspensions. These technologies are a relatively new approach to improve the effectiveness of particle size reduction and to reduce the time of processes. In general, they can be described as a combination of a bottom-up process (the building-up of particles) [15,16,17] followed by a top-down technology (disintegration) [18,19]. This method involves two particle size reduction steps. There is also a possibility for the combination of dry- and wet milling in one step, but literature data relating to the application of this combined method are lacking. Retsch GmbH (Haan, Germany) has recommended the combination of planetary ball milling, as dry milling, and pearl milling, as wet milling [20] techniques.

In wet micro- and nanonization, the application of additives is required in order to retain the individuality of the particles. Different additives are used to stabilize these particles: poly(vinylpyrrolidone) (PVP), Poloxamer^®^ (Poloxamer 188 = poly(ethylene)–poly(propylene glycol), polysorbate (Tween 80^®^ = poly(oxyethylenesorbitan monooleate)), Solutol^®^ (Solutol HS 15 = poly(ethylene glycol 15-hydroxystearate)), PVA (poly(vinyl alcohol)), *etc.* [21].

Milling and the presence of additives during milling have the ability to decrease the crystallinity of active materials [22,23]. The amorphous phase transition usually results in improved dissolution rates, thereby increasing the bioavailability, with the improvement being directly related to the extent of amorphization [24]. However, physical and chemical instability of amorphous material could be expected.

Meloxicam (MEL) is a non-steroidal anti-inflammatory drug (NSAID) with anti-inflammatory, analgesic and antipyretic effects. MEL was chosen as a model crystalline drug because of its poor aqueous solubility (4.4 µg/mL) [25] and relatively high melting point (270 °C) [26].

This research investigates the applicability of a wet milling method combining planetary ball and pearl milling. We set out to utilize particle size reduction to the micro- or nanometre range. The effects of milling time and the presence or absence of stabilizer (PVA) in reducing the particle size were investigated. The changes in crystallinity and morphology of MEL during milling have been studied. The effects of particle size reduction and amorphization on the dissolution rate were determined.

## 2. Results and Discussion

### 2.1. Effects of Milling Time on Particle size Distribution (PSD) and Specific Surface Area (SSA)

During our work, a combined wet milling technique (planetary ball mill and pearls, as milling media) was investigated. Analysis of the laser diffraction results revealed that the milling in the case of samples containing water as a dispersant medium resulted in a roughly 85% decrease in average MEL particle size. The D_10_, D_50_ and D_90_ values and the SSA are reported in Table 1.

After 10 min milling, micronized MEL was observed. However, further milling led to an increase in particle size, because MEL particles aggregated without the presence of PVA. The smallest average particle size (3.55 µm) and the highest SSA (1.89 m^2^/g) were obtained during the first 10 min of milling. During milling heat production was observed and the temperature of the samples increased up to 40–45 °C. The aqueous solubility of MEL (4.4 µg/mL, 25 °C) increased due to the elevated temperature (40 µg/mL, 37 °C), but it was not remarkable in our investigation (at about 0.04 *w*/*v*%), and 0.8 mg MEL could be dissolved in our dispersant medium (water) of the presence 2 g of active agent. The recrystallization of the small amount of dissolved MEL was irrelevant in terms of any particle size increase.

In the case of PVA-solution as a dispersant, after 10 min micronized, and after 50 min nanonized particles were noted, with monodisperse PSD (Table 2). The average particle size decreased efficiently, from approximately 35 µm to 126 nm, up to 50 min of milling. The SSA of MEL rose 150-fold compared to the raw MEL. Further milling led to only a slight decrease in particle size. PVA, used as a coating polymer helped the particles to separate from each other. Aggregation was also prevented, therefore the stability of the system could be improved.

### 2.2. Characterization of the Dried Products

#### 2.2.1. Physical Structure (XRPD and DSC)

For determination the effect of milling on the crystallinity of MEL pre-dispersions, samples milled for 10 min in water as a dispersant, and for 10 and 50 in PVA-solution as a dispersant were dried and characterized.

**Figure 1:**
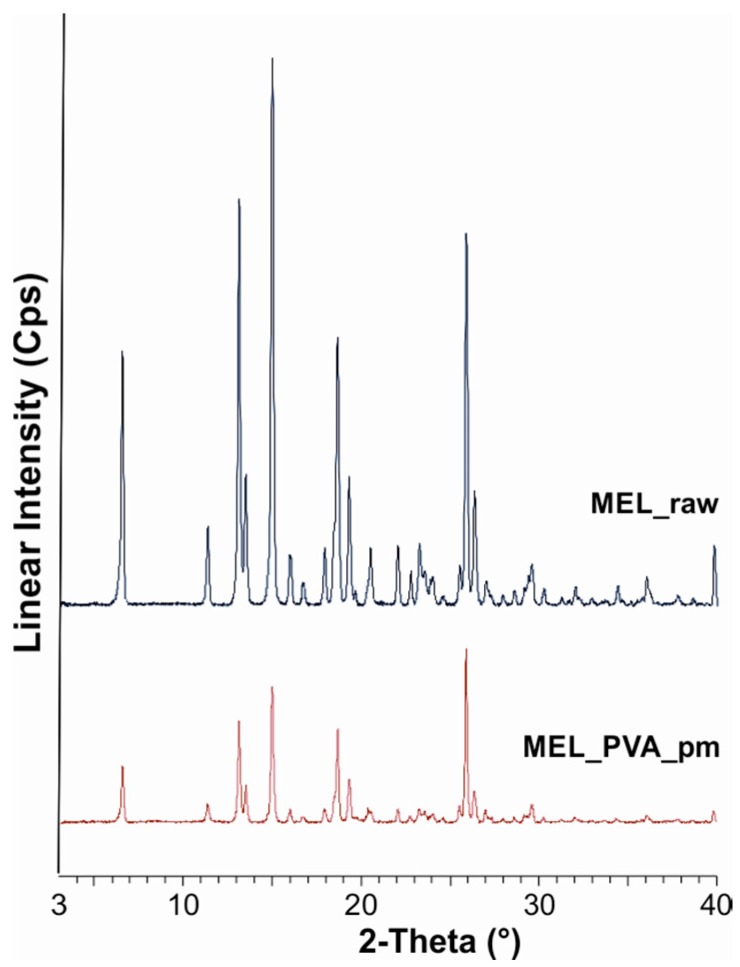
XRPD examination of MEL_raw and MEL_PVA_pm.

The XRPD diffractogram of raw MEL and of MEL in the physical mixture of MEL-PVA demonstrated its crystalline structure, as expected. The characteristic peaks are located at the following 2θ values: 13.22°, 15.06° and 26.46°. Ten min of milling in the water-containing sample caused the loss of the crystalline structure of the drug, and only 3% of the drug remained crystalline (Table 3). The intensities of the characteristic peaks were decreased in the case of the treated product (Figure 1). During the further milling aggregation was observed, because of the lack of PVA. At the end of milling (90 min), 9% of MEL remained crystalline.

**Figure 2:**
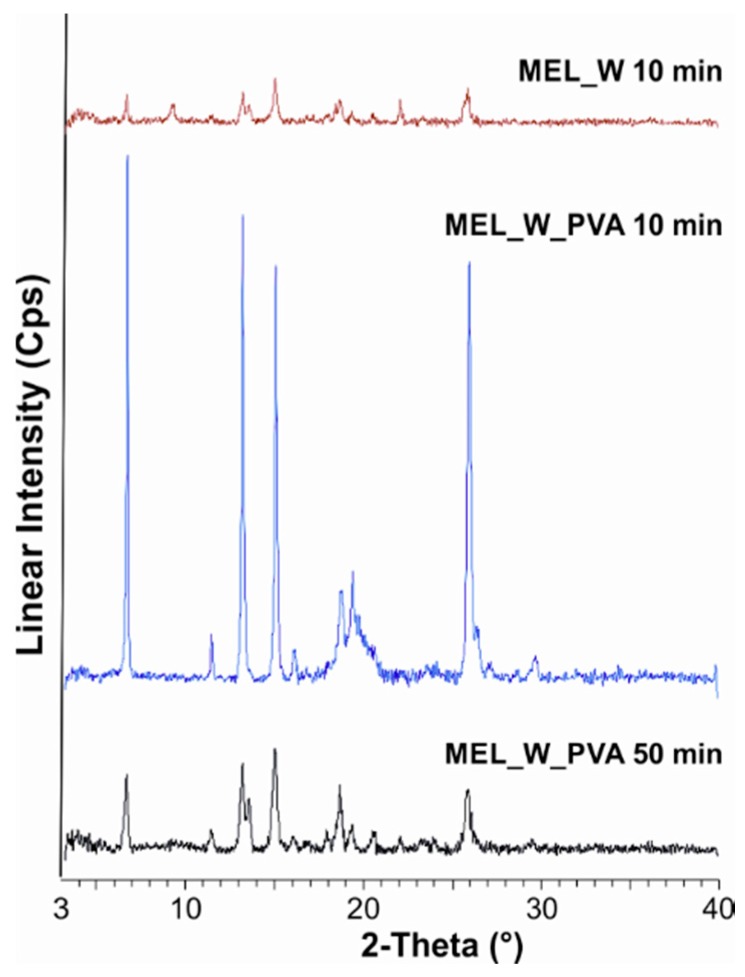
XRPD examination of MEL_W 10 min, MEL_W_PVA 10 min and MEL_W_PVA 50 min.

In the PVA-solution containing sample in the course of milling, a decrease in crystallinity was perceptible. After milling for 10 min, ~47% of the drug remained crystalline. After 90 min milling amorphization was detected, the degree of crystallinity was 2% (Table 3). The intensities of the characteristic peaks decreased due to the milling (Figure 2).

**Figure 3:**
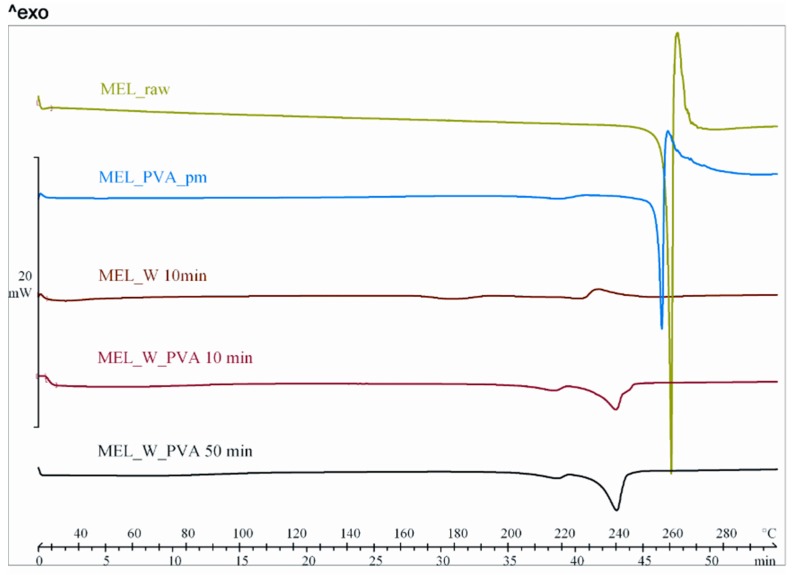
DSC curves of MEL_raw, MEL_PVA_pm, MEL_W 10 min, MEL_W_PVA 10 min and MEL_W_PVA 50 min.

DSC was employed to investigate the melting of MEL in the raw form, the physical mixture and in the milled dried products (Figure 3). The DSC curves of the raw MEL and of MEL in the physical mixture of MEL-PVA revealed a sharp endothermic peak at 259.1 and 255.9 °C, reflecting its melting point and confirming its crystalline structure. In case of water-containing samples the most significant decrease of crystallinity was observed after 10 min milling (peak: 227.0 °C). Increasing the milling time, aggregation was detected. Using PVA-solution as dispersant, DSC curves exhibited a broad endothermic peak for MEL, indicating that the crystallinity of the drug was decreased (Table 4). The residual MEL crystals in the products melted at a lower temperature than the crystals of raw MEL due to the smaller particle size and the increased degree of amorphization which was directly proportional to the duration of milling. This process was promoted by PVA, which was softened at 43 °C as glass transition temperature (*T_g_*) value.

It can be concluded that combined milling resulted in partial amorphization of MEL which could results in a higher dissolution rate compared to the raw drug and its physical mixtures.

#### 2.2.2. Particle Shape and Size

**Figure 4:**
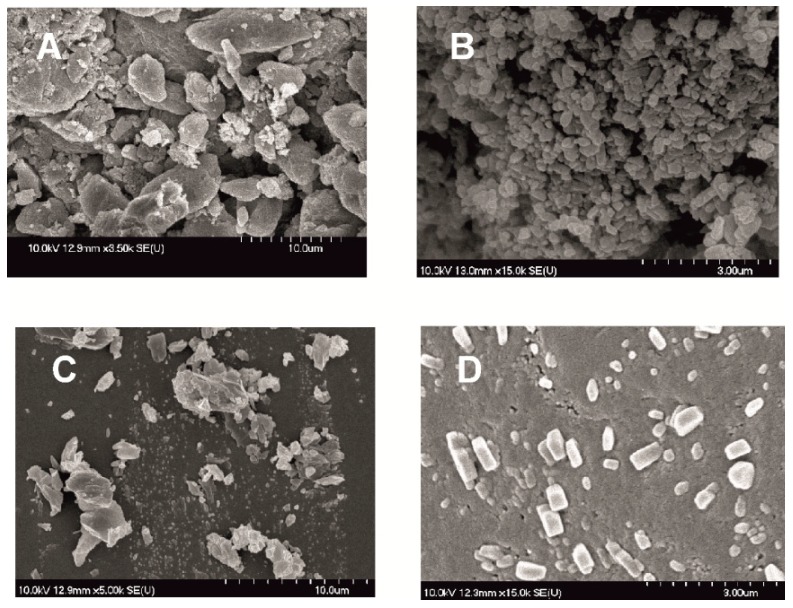
SEM pictures of MEL_raw (**A**); MEL_W 10 min (**B**); MEL_W_PVA 10 min (**C**) and MEL_W_PVA 50 min (**D**).

In the following SEM images (Figure 4) the morphology of the modified particles is demonstrated. Dried products produced by milled pre-dispersion in water for 10 min and MEL micronized and nanonized in the presence of PVA (10 and 50 min) were investigated. How the surface and shape changed after the milling process, compared with raw MEL, was checked. After taking samples after 10 min of milling in the case of water as a dispersant and 10 and 50 min of milling in the case of PVA-solution as the medium, the suspensions were dried and characterized. The raw MEL consisted mainly of angular, prismatic crystals with a broad size distribution. In the case of the water-containing samples, it was shown that micro-sized aggregates consisting of nanonized particles were formed. In the case of PVA-solution-containing sample, 10 min of milling resulted in irregular particle shapes, with the approximately 3 µm D_50_ value. MEL crystals could be detected between the amorphous PVA particles. After 50 min, nanonized MEL crystals (at about 120 nm) embedded in the PVA-film, with regular shape and smooth surface were perceived. The particle endurance during the treatment accounts for the smooth surfaces of the particles.

#### 2.2.3. *In Vitro* Dissolution Tests

**Figure 5:**
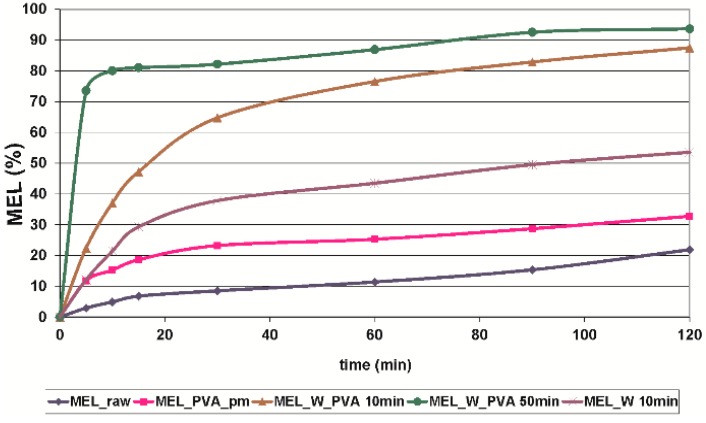
*In vitro* dissolution profile of MEL_raw, MEL_PVA_pm, MEL_W 10 min, MEL_W_PVA 10 min and MEL_W_PVA 50 min (SD < 0.05%).

The *in vitro* dissolution test was performed at pH 7.4, which could represent the media of the intestinal system where the absorption of MEL could be totally achieved. At pH 7.4 the conditions of the lung, nasal and skin epithelia could also be imitated. The *in vitro* dissolution showed the poor solubility and slow dissolution of MEL (D_50_ = 34.26 µm), and after 60 min approximately 10%, and after 2 h around 22% of the drug was liberated. Application of PVA (in the reference sample, where the size of the MEL did not decreased significantly) as hydrophilic wetting agent, could improve the dissolution rate by 2-fold. The micronization of the drug without PVA (D_50_ = 3.55 µm) could influence the dissolution rate 3-fold. Producing individual micronized (D_50_ = 2.96 µm) and nanonized (D_50_ = 0.126 µm) MEL drug particles in the presence of PVA, the extent of dissolution was enhanced significantly by the increased specific surface area. The amorphous character and the submicron range of the drug resulted in a very fast dissolution, with more than 70% of MEL liberated after the first 5 min (Figure 5).

## 3. Experimental Section

### 3.1. Materials

Meloxicam (4-hydroxy-2-methyl-*N*-(5-methyl-2-thiazolyl)-2*H*-benzothiazine-3-carboxamide-1,1-dioxide) was obtained from EGIS Ltd. (Budapest, Hungary). The grinding additive, PVA (polyvinyl alcohol), was purchased from Gedeon Richter Plc. (Budapest, Hungary).

### 3.2. Methods

#### 3.2.1. Combined Milling

A wet milling technique (a combination of planetary ball and pearl milling) was employed. The drug (2 g) was first suspended in the dispersant medium (18 g), consisting of water and aqueous solution of PVA (the use of lower concentrations of PVA led to extensive aggregation of the MEL particles because of the lesser effectiveness in overcoming the cohesive forces). The resulting suspensions (10% drug content) were wet milled at 400 rpm in the milling chamber (50 mL) of a planetary ball mill (PM 100 MA, Retsch GmbH, Haan, Germany). The milling balls were 0.3 mm ZrO_2_ beads. As a preliminary experiment, the effects of different pearl weights (10, 20, 50 and 150 g) on the particle size reduction were investigated, and milling was also carried out without pearls as a benchmark. The most effective milling was observed when 20 g of pearls was applied. Increasing the weight of pearls did not result in further particle size reduction, therefore 20 g of beads were used for further experiments. The milled suspensions were separated with a 150 µm mesh size sieve. Suspension sampling was carried out at milling times of 10, 20, 30, 40, 50, 60, 70, 80 and 90 (end of milling) min to perform the particle size analysis.

#### 3.2.2. Determination of Particle Size Distribution and Specific Surface Area by a Laser Diffraction Method

The volume based particle size distribution (PSD) of the raw MEL was measured by laser diffraction (Mastersizer 2000, Malvern Instruments Ltd., Worcestershire, UK) with the following parameters: 300RF lens; small volume dispersion unit (2500 rpm); refractive index for dispersed particles 1.720; refractive index for dispersion medium 1.330. The Dynamic Laser Light Scattering method was used to determine the PSD. The particle size of the MEL was determined directly on the initial suspension in water in which PVA was dissolved. The size analysis was repeated three times. Water was used as dispersant medium and the obscuration was in the range 11%–16% for all measurements. In all cases, the volume weighted particle size distributions, D_10_, D_50_, and D_90_ (where for example D_50_ is the maximum particle diameter below which 50% of the sample volume exists–also known as the median particle size by volume) were determined and evaluated. The specific surface area was derived from the particle size distribution data. The assumption was made that all the measured particles were spherical.

#### 3.2.3. Preparation of Solid Products for Physical-Chemical Investigations of Products

After the particle size determination, the selected pre-dispersions were dried in a vacuum dryer (Binder GmbH, Tuttlingen, Germany) at 40 °C in order to obtain solid products for physicochemical investigations. The abbreviations of solid state samples are summarized in Table 5.

#### 3.2.4. Further Investigations of the Products

##### Structural Analysis

The physical state of the MEL in the samples was evaluated by XRPD. XRPD patterns were produced with a Bruker D8 Advance diffractometer (Bruker AXS GmbH, Karlsruhe, Germany) system with Cu K λI radiation (*λ* = 1.5406 Å). The samples were scanned at 40 kV and 40 mA from 3° to 40° 2θ, at a scanning speed of 0.05°/s and a step size of 0.010°. The crystallinity (*X_c_*) of the MEL in dried pre-dispersions, milled for 10, 50, 60 and 90 min was determined semi-quantitatively in case of both dispersant medium via the mean of the decrease of the total area beneath the curve of 3 characteristic peaks (*A_crystalline_*) compared to the raw MEL and MEL–poly(vinyl alcohol) physical mixture (MEL_PVA_pm) (*A_crystalline_ + A_amorphous_*):

(1) $$X_{c}=\frac{A_{crystalline}}{A_{crystalline}+A_{amorphous}}\times 100$$

DSC measurements were carried out with a Mettler Toledo DSC 821^e^ thermal analysis system equipped with the STAR^e^ thermal analysis program V9.0 (Mettler Inc., Schwerzenbach, Switzerland). Approximately 2–5 mg of pure drug or product was examined in the temperature range between 25 °C and 300 °C. The heating rate was 5 °C·min^−1^. Argon was used as carrier gas at a flow rate of 10 L·h^−1^ during the DSC investigations.

##### Image Analysis (SEM)

The shape and surface characteristics of the samples were visualized by using a scanning electron microscope (Hitachi S4700, Hitachi Scientific Ltd., Tokyo, Japan). The samples were sputter-coated with gold–palladium under an argon atmosphere, using a gold sputter module in a high-vacuum evaporator and the samples were examined at 15 kV and 10 μA. The air pressure was 1.3–13 MPa. The milling-produced suspensions were dried in order to obtain solid products for SEM analyses.

##### *In Vitro* Release

The paddle method with the USP dissolution apparatus (USP dissolution apparatus, type II Pharma Test, Heinburg, Germany) was used to examine MEL (1.67 mg), MEL_PVA_pm and the products (2.0875 mg). The medium was 100 mL of phosphate buffer of pH 7.4 ± 0.1. The basket was rotated at 100 rpm and sampling was performed up to 120 min. After filtration (filtration pore size 0.22 µm; applying a Millex-HV syringe-driven filter unit, Millipore Corporation, Bedford, MA, USA) and dilution, the MEL contents of the samples were determined by spectrophotometry (ATI-UNICAM UV/VIS Spectrophotometer, Cambridge, UK) at 362 nm.

## 4. Conclusions

A combination of planetary ball and pearl milling (using pearls as milling media) can be applied as a wet milling procedure to decrease the particle size and change the crystal morphology of MEL. Wet milling in aqueous medium adheres to green technology principles as the product does not contain organic solvent residues. Besides several advantages of wet milling, it is necessary to calculate the wear of pearls during milling.

During our work at constant rotation rate in the presence and absence of a stabilizer, the effects of milling time on the particle size reduction was determined (Table 6). Without additive, in the case of water-containing samples micronization could be achieved (D_50_ = 3.55 µm). In the presence of PVA, depending on the milling time, the particle size of the drug could be reduced to the micro-(after 10 min), (D_50_ = 2.96 µm) or nanometre range (after 50 min, D_50_ = 126 nm). The effect of milling on the crystallinity of MEL was investigated. XRPD and DSC examinations revealed a decrease in the crystallinity of MEL. In the case of water-containing samples (without PVA) aggregation occurred during the course of milling. In the PVA-containing samples amorphization was determined (the degree of MEL crystallinity was 2% at the end of the milling at 90 min). SEM images revealed the aggregation of nanosized particles in water-containing samples. In the presence of additive milling for 10 min resulted in irregularly shaped particles. The nanonized MEL crystals exhibited a regular shape and smooth surface. The *in vitro* dissolution tests showed that the reduction of the particle size of MEL, the increased SSA and the structural transformation of drug resulted in a rapid dissolution in case of nanonized MEL-containing product. The amorphous form of the drug does not require lattice energy to break the bonds during the dissolution process as in the crystalline state case.

The combined wet milling technology was suitable for preparation of micronized MEL without the use of stabilizer and, depending on the milling time, of micronized and nanonized drug particles-containing pre-dispersions in the presence of PVA. Milling in the presence of additive could be the first step of pre-formulation and further formulation procedures. Decreased particle size (especially accessing the nanosize range) and the amorphization of drug could ensure higher dissolution rate and better bioavailability of poorly-water soluble drugs, though instability problems could occur in the case of amorphous forms of materials. To check the stability of the systems further investigations are needed.

Because of the low need for dispersant medium, the combined method can be used for efficient milling, and it is also suggested for the preparation of the pre-dispersions with micro- and nanosized particles, and recommended for the development of particle size-controlled therapeutic systems.

## Figures and Tables

**Table 1:** MEL PSD and SSA in pre-dispersions containing water as dispersant.

Milling Time (min)	D_10_ (µm)	D_50_ (µm)	D_90_ (µm)	SSA (m^2^/g)
raw MEL	11.40	34.26	73.59	0.332
10	1.76	3.55	8.69	1.89
20	2.63	5.74	14.21	1.22
30	2.62	5.99	18.97	1.18
40	2.77	6.84	24.54	1.07
50	2.72	6.13	19.56	1.15
60	2.57	6.12	20.87	1.18
70	2.77	7.13	26.26	1.05
80	2.58	5.94	22.54	1.19
90	2.51	5.55	18.88	1.25

**Table 2:** MEL PSD and SSA in pre-dispersions containing PVA-solution as dispersant.

Milling Time (min)	D_10_ (µm)	D_50_ (µm)	D_90_ (µm)	SSA (m^2^/g)
raw MEL	11.40	34.26	73.59	0.332
10	0.24	2.96	29.40	7.71
20	0.084	0.169	2.863	34.6
30	0.090	0.337	4.180	27.2
40	0.074	0.134	1.358	46
50	0.074	0.126	0.253	49.5
60	0.072	0.122	0.219	52
70	0.070	0.121	0.225	52.6
80	0.072	0.119	0.209	53.2
90	0.064	0.116	0.219	57.9

**Table 3:** The degree of MEL crystallinity (%) for different dispersant media and milling times.

Sample	Crystallinity (%)
MEL_raw	100
MEL_PVA_pm	80
MEL_W 10 min	3
MEL_W 50 min	5
MEL_W 60 min	8
MEL_W 90 min	9
MEL_W_PVA 10 min	48
MEL_W_PVA 50 min	12
MEL_W_PVA 60 min	5
MEL_W_PVA 90 min	2

**Table 4:** Thermoanalytical evaluation of MEL_raw, MEL_PVA_pm and dried milled products.

Parameters	Raw MEL	pm	Dried MEL_W Samples				Dried MEL_W_PVA Samples			
**Milling time (min)**	**-**	**-**	**10**	**50**	**60**	**90**	**10**	**50**	**60**	**90**
∆H * (J/g)	−148.5	−29.9	−7.5	−40.1	−64.4	−40.8	−60.3	−70.9	−32.4	−46.4
**Onset (°C)**	258.4	235.9	217.9	233.5	248.5	247.1	231.6	232.9	207.1	206.2
**Peak (°C)**	259.1	255.9	227.0	238.7	250.7	250.2	239.8	240.1	216.7	217.4
**Endset (°C)**	261.0	255.9	230.0	240.4	250.8	251.6	243.1	243.2	222.3	222.3

* ∆H: normalized integral.

**Table 5:** Sample abbreviations.

Sample Name	MEL (%)	PVA (%)	Description
MEL_raw	100	-	untreated MEL
MEL_PVA_pm	80	20	physical mixture of MEL and PVA
MEL_W 10 min	100	-	dried MEL, milled for 10 min in water
MEL_W 50 min	100	-	dried MEL, milled for 50 min in water
MEL_W 60 min	100	-	dried MEL, milled for 60 min in water
MEL_W 90 min	100	-	dried MEL, milled for 90 min in water
MEL_W_PVA 10 min	80	20	dried product of milling for 10 min in PVA-solution
MEL_W_PVA 50 min	80	20	dried product of milling for 50 min in PVA-solution
MEL_W_PVA 60 min	80	20	dried product of milling for 60 min in PVA-solution
MEL_W_PVA 90 min	80	20	dried product of milling for 90 min in PVA-solution

**Table 6:** Summarized information about the determined properties of the samples.

Sample	D_50_ (µm)	Crystallinity (%)	Dissolved MEL at 30 min (%)
MEL_raw	34.26	100	8.53
MEL_PVA_pm	20.15	80	29.22
MEL_W 10 min	3.55	3	37.82
MEL_W_PVA 10 min	2.96	48	64.73
MEL_W_PVA 50 min	0.126	12	82.16

## References

[1] Truong-Dinh Tran T., Tran K.A., Ha-Lien Tran P. (2015). Modulation of particle size and molecular interactions by sonoprecipitation method for enhancing dissolution rate of poorly water-soluble drug. Ultrason. Sonochem..

[2] Mansouri S., Kralj T.P., Morton D., Chen X.D., Woo M.W. (2014). Squeezing out ultrafine hydrophobic and poor water-soluble drug particles with water vapour. Adv. Powder Technol..

[3] Caliandro R., di Profio G., Nicolotti O. (2013). Multivariate analysis of quaternary carbamazepine–saccharin mixtures by X-ray diffraction and infrared spectroscopy. J. Pharm. Biomed. Anal..

[4] Miclea L.M., Vlaia L., Vlaia V., D.I. Hădărugă C. (2010). Mircioiu, Preparation and characterization of inclusion complexes of meloxicam and α-cyclodextrin and β-cyclodextrin. Farmacia.

[5] Verma S., Gokhale R., Burgess D.J. (2009). A comparative study of top-down and bottom-up approaches for the preparation of micro/nanosuspensions. Int. J. Pharm..

[6] Serajuddin A.T.M. (2007). Salt formation to improve drug solubility. Adv. Drug Deliv. Rev..

[7] Hassan M.A., Suleiman M.S., Najib N.M. (1990). Improvement of the *in vitro* dissolution characteristics of famotidine by inclusion in β-cyclodextrin. Int. J. Pharm..

[8] Paulino A.S., Rauber G., Campos C.E.M., Maurício M.H.P., de Avillez R.R., Capobianco G., Cardoso S.G., Cuffini S.L. (2013). Dissolution enhancement of deflazacort using hollow crystals prepared by antisolvent crystallization process. Eur. J. Pharm. Sci..

[9] Lim R.T.Y., Ng W.K., Tan R.B.H. (2013). Dissolution enhancement of indomethacin via amorphization using co-milling and supercritical co-precipitation processing. Powder Technol..

[10] Liu P., Rong X., Laru J., van Veen B., Kiesvaara J., Hirvonen J., Laaksonen T., Peltonen L. (2011). Nanosuspensions of poorly soluble drugs: Preparation and development by wet milling. Int. J. Pharm..

[11] Noyes A.A., Whitney W.R. (1897). The rate of solution of solid substances in their own solutions. J. Am. Chem. Soc..

[12] Manfredini & Schianchi MS DRYTECH: Continuous Evolution in the Dry Preparation of Raw Materials. http://www.manfredinieschianchi.com/406-2EN-advantages-of-dry-grindiing.htm.

[13] Swarbrick J. (2013). Encyclopedia of Pharmaceutical Technology.

[14] Merisko-Liversidge E., Liversidge G.G. (2011). Nanosizing for oral and parenteral drug delivery: A perspective on formulating poorly-water soluble compounds using wet media milling technology. Adv. Drug. Deliv. Rev..

[15] Blagden N., Matas M., Gavan P.T., York P. (2007). Crystal engineering of active pharmaceutical ingredients to improve solubility and dissolution rates. Adv. Drug Deliv. Rev..

[16] Bund R.K., Pandit A.B. (2007). Sonocrystallization: Effect on lactose recovery and crystal habit. Ultrason. Sonochem..

[17] Bakar M.R.A., Nagy Z.K., Saleemi A.N., Rielly C.D. (2009). The impact of direct nucleation control on crystal size distribution in pharmaceutical crystallization processes. Cryst. Growth Des..

[18] Salazar J., Ghanem A., Müller R.H., Möschwitzer J.P. (2012). Nanocrystals: Comparison of the size reduction effectiveness of a novel combinative method with conventional top-down approaches. Eur. J. Pharm. Biopharm..

[19] Möschwitzer J.P. (2013). Drug nanocrystals in the commercial pharmaceutical development process. Int. J. Pharm..

[20] Direct Industry, Retsch^®^: All Retsch Catalogues and Technical Brochures. http://pdf.directindustry.com/pdf/retsch/the-sample-high-energy-ball-mills/19308-518973.html.

[21] Paltonen L., Hirvonen J. (2010). Pharmaceutical nanocrystals by nanomilling: Critical process parameters, particle fracturing and stabilization methods. J. Pharm. Pharmacol..

[22] Smith G., Hussain A., Bukhari N.I., Ermolina I. (2015). Quantification of residual crystallinity in ball milled commercially sourced lactose monohydrate by thermo-analytical techniques and terahertz spectroscopy. Eur. J. Pharm. Biopharm..

[23] Mártha C., Kürti L., Farkas G., Jójárt-Laczkovich O., Szalontai B., Glässer E., Deli M.A., Szabó-Révész P. (2013). Effects of polymers on the crystallinity of nanonized meloxicam during a co-grinding process. Eur. Polym. J..

[24] Pan X., Julian T., Augsburger L. (2008). Increasing the dissolution rate of a low-solubility drug through a crystalline-amorphous transition: A case study with indomethacin. Drug Dev. Ind. Pharm..

[25] Ambrus R., Kocbek P., Kristl J., Šibanc R., Rajkó R., Szabó-Révész P. (2009). Investigation of preparation parameters to improve the dissolution of poorly water-soluble meloxicam. Int. J. Pharm..

[26] Hughey J.R., Keen J.M., Brough C., Saeger S., McGinity J.W. (2011). Thermal processing of a poorly water-soluble drug substance exhibiting a high melting point: The utility of KinetiSol^®^ Dispersing. Int. J. Pharm..

